# Displacements of the Uterus

**Published:** 1875-09

**Authors:** 


### Selections.

### DISPLACEMENTS OF THE UTERUS.

### MEDICAL AND SURGICAL SOCIETY OF BALTIMORE.

Dr. Erich introduced the subject by an able paper, which was published in the June number of the *Physician and Surgeon.*

*Dr. Arnold.* It is somewhat singular that there prevail the most divergent views respecting the indications for the mechanical treatment of uterine displacements. While, on the other hand, many eminent gynecologists speak in the highest terms of the benefit derived from the use of pessaries, there are others who deprecate the employment of these contrivances altogether, or would confine them to cases of procidentia and prolapsus uteri. This prejudice against the use of pessaries has undoubtedly been fostered by their ill effects or inefficiency in many instanees; though it cannot be denied that errors of diagnosis, inexpertness, or a want of proper attention, may justly be accused as the cause of want of success. Still it is now generally conceded that anteversions and retroversions very rarely require any special treatment, as these instances of displacement seldom give rise to notable symptoms; and besides, a considerable proportion of these deviations result from morbid causes, such as subinvolution, pelvic exudations, tumors, etc., which must be met by appropriate remedial measures, the uterus being only secondarily involved. It appears to me that the only cases of displacement which suggest mechanical interference are those that are accompanied by dysmenorrhœa or chronic uterine catarrh, and I am inclined to believe, from actual experience, that retroflexion is by far the most frequent cause of these troublesome affections. Whatever objections may be advanced against the too exclusive use of mechanical means to correct abnormal positions of the uterus, it cannot be denied that a retroflexed uterus produces such significant symptoms that urgently indicate some extra or intra-uterine support for their relief. I cannot recollect that I have ever seen well-marked cases of painful menstruation or difficulty of micturition or defecation that resulted from mere anteversion or retroversion, which hardly interferes with the accommodating positions of the uterus; but it can readily be understood why the traction and pressure caused by retroflexion must cause various and highly troublesome derangements. Much has been said respecting the special morbid conditions that lead to and keep up the backward displacement of the womb, yet it is undecided whether the attachments of the uterus to the adjacent structures that retain it in its abnormal position precede or follow this occurrence. Retroflexions may be practically divided into those which are due to firm adhesions, and into such which depend on the relaxation of the uterine supports. Tumors of the posterior wall of the uterus would, of course, constitute a separate class. Well adjusted pessaries would, undoubtedly, promise good results in cases where the uterus is not bound down by rigid attachments; though I must confess that in my hands, even in this form of displacement, the use of pessaries has had but a limited success.

*Dr. Erich.* Uterine displacements, of whatever nature, as long as they produce no discomfort or sterility, should not be interfered with. If Dr. Arnold has never seen a case of anteversion or retroversion producing unpleasant symptoms, his experience has been different from mine, or that of any gynecologist with whom I am acquainted. Anteversion as well as retroversion, being impossible without preceding descent of the uterus, is frequently accompanied by the characteristic symptoms of prolapsus uteri, as painful sensations of weight and dragging in the pelvis, rectal and vesicular irritation, etc., all which symptoms are increased when the patient assumes the erect position, and diminished upon assuming the horizontal position. Without discussing the question whether congestion, inflammation and subinvolution of the uterus are the cause or the effect of its displacement, it is certain that none of these affections can be treated successfully without a proper reduction of the displacement; by which means we restore the more or less impeded uterine circulation to its normal condition, and relieve the traction made upon the uterine nerves.

*Dr. Noel.* I wish to ask some questions which have a practical bearing on this subject. Can rheumatic or other trouble of the round ligament on one side produce tilting of the womb? Do retroflexion and anteflexion produce much trouble if there be no inflammation? Can anteversion or retroversion be produced by over-loaded rectum or distended bladder? In retroflexion, is there an atrophy of the posterior wall of the neck, and of the anterior wall in anteflexion? Do the uterine walls atrophy primarily? In prolapsed ovary what is the best method of diagnosis? The opinions we hold on these points must have a great effect on our treatment of uterine displacements.

*Dr. Erich.* Any disease of the round ligaments causing much shortening of the same, may cause a slight change in the position of the uterus; it can, however, not produce anteversion, because the insertion of these ligaments into the uterus is below the upper margin of the pubes, over which they pass. Great shortening of these ligaments would cause the fundus to be lifted up higher than natural, which is the opposite of what takes place in anteversion.

Retroflexion and anteflexion are very apt to produce obstructive dysmenorrhœa and sterility, whether inflammation is present or not:

I am fully convinced that an over-loaded rectum or distended bladder cannot produce retro or anteversion or flexion. The rectum descends in the pelvis a little to the left of the uterus, and upon being distended it would push the uterus bodily to the right side of the pelvis without the slightest possibility of producing anteversion or retroflexion. A distended bladder might push the uterus bodily into the hollow of the sacrum, but could never produce retroversion or anteflexion. Authors who have advocated the opposite view (as, for instance, Virchow, who thinks that anteflexion may be caused by a distended bladder,) have surely not reasoned out their theory while standing before the dead subject.

In flexions of long standing there is sure to be atrophy upon the concave surface of the flexed uterus, which may be primary or not, according to the cause producing the flexion.

The best method of diagnosis in prolapsed ovary consists in dilating the urethra and inserting the finger of one hand into the bladder, and that of the other into the rectum.

*Dr. Morris.* Dr. Erich lays too much stress on the mechanical treatment, and makes but little mention of the therapeutical. Dr. Atlee, of Philadelphia, says that he can cure ten cases with medicine to two with pessaries. Anteversion is the normal position of the virgin uterus. Displacements are nothing in themselves; only symptoms or evidences of disease. I have seen virgins suffer from anteversion with all the symptoms mentioned by authors, but these cases were the result of accident, general impairment of the health, or secret vice, and no pessary can have had any influence in relieving them. In prolapse and procidentia uteri, pessaries are the best treatment, and Dr. Erich's is a very excellent one for the purpose. I have used one like it for seven years, and the results have been good. Lateral displacements are almost impossible, except from inflammation of the ovary or cellular tissue, and I am disposed to think that they do not exist except from these causes. It is fashionable to talk about displacement of the ovary. I had a patient with some obscure uterine trouble, which I was unable to cure. I took her to New York; Dr. Fordyce Barker thought there might be displaced ovary, but was not certain; Drs. Sims and Nott could not detect it; Dr. Nott thought the coccyx produced the trouble. She had a slight fissure of the rectum, which was cured, but to-day she is in the same condition she was seven years ago. It is a very difficult thing to diagnose displacement of the ovary. I have never been able to detect the ovary through the rectum, but I have found the uterus engorged. Has Dr. Noel ever made out falling of the ovary?

*Dr. Noel.* Yes; in two cases. The patients were thin, and by the fingers in the vagina, strong pressure being made in the flank, I was able to feel the ovary, and roll it between my fingers. The patients complained of faintness, etc., as when the testicle is pressed.

*Dr. Morris.* They will complain of the same sensations when applications are made to the os. In women who have had no children the cervix is more likely to be the part diseased; in multiparous, the body. Sterile women give us a great deal of trouble. They have lucorrhœa (probably from some abrasion), some uterine enlargement, and general bad health, and we usually fail to relieve them. Baker Brown recommends slitting the cervix and keeping it open, hoping that they may become pregnant. Disease of the mucous or serous surfaces, or of the parenchyma, will produce enlargement of the womb. If there be no enlargement, an arrest of the circulation, as described by Dr. Erich, is impossible. In the treatment of these cases I have great faith in depletion, and it is my main remedy. I do not use the leech, because it is troublesome to apply, but prefer the artificial leech. Two or three ounces of blood can be drawn without trouble, and as soon as the engorgement is relieved the uterus takes its normal position. Muriate of ammonia gr. xv-xx t. d.; hip bath, cold douche, etc., are useful. Large cotton pessaries, soaked in glycerine, answer all the indications usually sought to be fulfilled by the hard pessary, and produce no injury. It is important to keep the rectum unloaded, and I use for this purpose a dinner pill containing Barbadoes aloes gr. ij, ext. nucis vom. gr. $\frac{1}{2}$, ext. rhei. gr. j, gum mastich q. s. Abdominal supporters I think are a great nuisance; only useful in fat women with pendulous abdomen; in thin women they do harm.

*Dr. Erich.* I agree that harm has been done by the injudicious use of pessaries, and if they were entirely abolished the world would lose nothing, because the evil done by them in inexperienced hands probably more than equals the good produced when skillfully applied. But we must not condemn a remedy because it is capable of doing harm when used by the ignorant. Harm has been done by splints, mercury, and many other articles, but we do not condemn the remedy, only the ignorant use of it. For a general practitioner to treat displacements successfully he must acquaint himself thoroughly with the anatomy of the parts, and be a good mechanic to adapt his means to the ends to be accomplished. How do pessaries relieve displacement? In varicose ulcers we find that elevation of the limb does good by relieving the pressure of the blood and giving rest. So a pessary elevates the displaced organ, relieves venous congestion by removing the impediment to the circulation, gives it rest and prevents motion. The pessary used should be flexible, easily adjustable and painless. If it produces pain, remove it at once and try another. In sterility with displacement, the simple correction of this will often be sufficient. Retroversion or anteversion must be preceded by descent. This descent can be relieved by pessaries which, by holding the womb in its normal position, allow the relaxed ligaments to contract, and, if the cause can be removed, the case in a few months can be cured. If the utero-sacral ligaments are short and there be much pressure from above, anteflexion will be produced. Another cause of flexion in the lower third is crowding the os of an anteverted womb into the hollow of the sacrum. Anteflexion in virgins only requires treatment when it produces dysmenorrhoea. Then dilate the neck by sponge-tents, and keep it open by means of an intra-uterine stem. This treatment, if persevered in for two or three months, will relieve the pain at the menstrual period.

*Dr. Arnold.* The modern practice of interference with the uterus is the result of efforts to relieve dysmenorrhoea, in which the older practitioners were not successful. This has produced our race of gynecologists. To them we owe many important results. Among fifty cases of disease of the womb which come to me for treatment, forty-five will be suffering from dysmenorrhoea. The vast majority of these are not cured either by general treatment or mechanical appliances. General or local treatment has a certain proportion of suecess, but there are many cases in which all treatment seems to be of but little efficacy. I have come to the conclusion that the pain is produced by the same cause that produces the pain in parturition.—*Baltimore Physician and Surgeon.*

